# Recent Developments in the Chemistry of Deoxyribonucleic Acid (DNA) Intercalators: Principles, Design, Synthesis, Applications and Trends 

**DOI:** 10.3390/molecules14051725

**Published:** 2009-05-07

**Authors:** Brenno A. D. Neto, Alexandre A. M. Lapis

**Affiliations:** 1 Laboratory of Medicinal and Technological Chemistry, University of Brasilia (IQ-UnB), Brasilia, DF, Brazil; 2 Universidade Federal do Pampa, Unipampa, Bagé, RS, Brazil

**Keywords:** DNA, RNA, photointercalators, fluorescence, rational design, therapy, cancer

## Abstract

In the present overview, we describe the bases of intercalation of small molecules (cationic and polar neutral compounds) in DNA. We briefly describe the importance of DNA structure and principles of intercalation. Selected syntheses, possibilities and applications are shown to exemplify the importance, drawbacks and challenges in this pertinent, new, and exciting research area. Additionally, some clinical applications (molecular processes, cancer therapy and others) and trends are described.

## 1. Introduction

DNA is a nucleic acid (biomolecule) that contains the genetic instructions specifying the biological development of all cellular forms of life (and many viruses). DNA is often referred to as the molecule of heredity, as it is responsible for the genetic propagation of all traits [1,2,3]. During reproduction, DNA is replicated and transmitted to the offspring. Its sequence defines many features ranging from organism type through physical traits to disease susceptibility. As it is nowadays well-established, the DNA sequence is copied (transcription) onto RNA biomolecules, which are then used in protein synthesis to encode a specific protein sequence (translation). For instance, understanding on a molecular level how genetic information is expressed and how to stimulate or prevent gene expression is a key step toward the development of new chemotherapeutic strategies. It is of great interest considering that several genetic sequences of many organisms are now known (and in particular, the human genome is known). Much effort has gone into establishing ways to control specific gene expression, as a way to prevent many diseases. 

Without doubt, (bio)chemical sensor technologies that focus on the direct detection of nucleic acids (DNA and RNA) are currently an area of tremendous interest, as they play a major role in forensics [4], pharmaceutical applications [5], medical diagnosis [6], genetic screening [7], rational drug design [8], diagnosis of drug resistance [9], food and agricultural analysis [10], environmental control [11], and bioterrorism prevention [12], among others [13]. In this sense, the understanding of the principles that rule this new and exciting field of research is of great importance [14] for the rational design, synthesis and applications of new DNA intercalators. 

There are several types of sensors, including organometallic complexes, neutral structures, electrochemical sensors, acoustical and optical sensors among others [6,15,16]. DNA photointercalator sensors are potentially a very powerful tool for quality-control testing of different kinds of products through nucleic acid technology [17]. They might also be used to analyze many products for the presence of toxins and pathogens, antibiotics, pesticides and chemicals. The development of novel, sensitive and selective sensors for the detection of DNA polynucleotides (PNs) has become a very active research field in recent years. For instance, the use of DNA photointercalators can help us to gather information on how these biomolecules are involved in the processes within the cells. The direct visualization of nucleic acids *in vivo* can provide information about the location, kinetics and function of these biomolecules, playing a major role in the understanding of different inter- and intracellular processes [18]. For instance, one of the main characteristics of a photointercalator to be used in quantitative real-time polymerase chain reaction assays (qRT-PCR) is that it must not affect the DNA-polymerase thermostable enzyme activity. Likewise, techniques such as PCR require probes with sufficient sensitivity to detect very small amounts of samples quantitatively [6] and, in some cases, selectivity must be adequate to identify a specific PN sequence [19]. In general, photointercalators probes function by binding to PNs by hydrophobic or electrostatic interactions that are nonspecific. The nonspecific binding means that those probes are capable of binding to PNs irrespective of their sequence. Such kinds of probes provide information about the amount of PNs available in a sample or cell, and even their position. Nevertheless, they usually are not specific to a definite target sequence. 

Fluorescence is widely used because it is by far the most sensitive of the available spectroscopic techniques [20]. In view of this, the development of DNA intercalators that display a “light up” effect (increase on its fluorescence intensity upon binding) or, in some cases “light off”, is mandatory to the progress of this field of knowledge. Typically, fluorescence requires micromolar concentrations of the intercalator and DNA, while NMR requires millimolar concentrations. DNA exhibits some intrinsic fluorescence, but the emission is too weak, and too deep in the ultra-violet spectrum for practical emission applications [21]. Mass and tandem mass spectrometry are also very useful techniques to study the intercalation, however, information such as DNA base sequence of intercalation may not be so direct. The use of liquid chromatography-tandem mass spectrometry to the analysis of reactive drug metabolites (and fluorescent metabolites) may be a viable alternative of analysis [22]. Electrochemical methods can also be very sensitive and useful technique, mainly because it is possible obtain to sequence recognition, as it has been recently reviewed [23].

In the present overview, we intend to describe the basis of DNA photointercalator technology, recent developments in the molecular architecture and rational design of small organic (and/or organometallic) photointercalator probes, their synthesis, applications and possibilities to be applied, and perspectives of research in the field of photointercalators. 

## 2. DNA structure: a basic background

**Figure 1:**
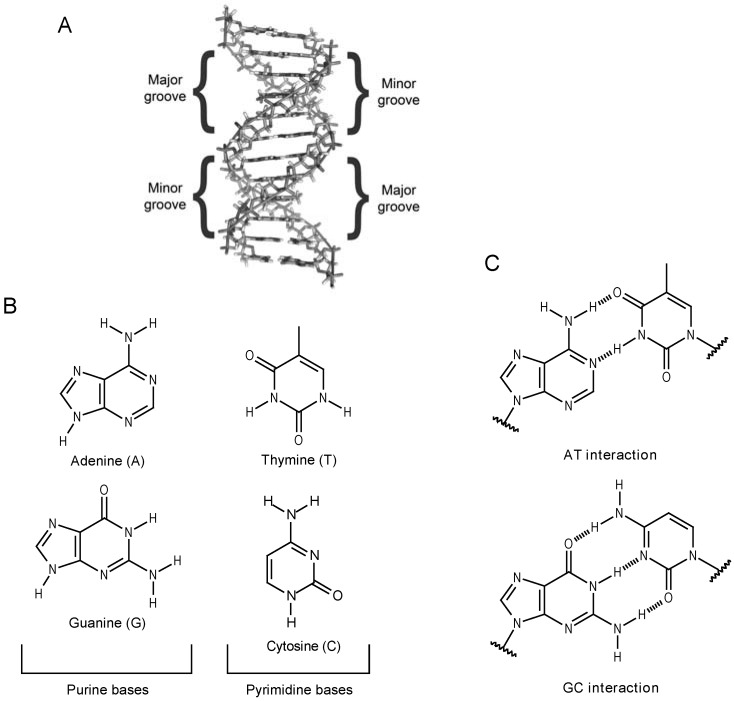
DNA basic 3D structure and bases interactions: (A) DNA and its representative form. Note major and minor grooves indication in the DNA structure; (B) Purine (A and G) and pyrimidine (T and C) bases; (C) Complementary base-pairs interactions (hydrogen bonding).

Double strand DNA (dsDNA, Figure 1-A) is a structure which displays an antiparallel double helix held together by hydrogen-bonding interactions between complementary base-pairs (Figure 1-B): adenine (A), thymine (T), guanine (G) and cytidine or cytosine (C), where it is possible to observe CG and TA interactions (Figure 1-C). A and G are purine bases, while T and C are pyrimidine bases. Although unusual, DNA can also be found as single strands (ssDNA). However, in biological systems, DNA is found as dsDNA.

**Figure 2:**
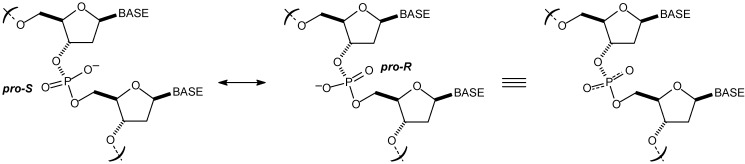
Stereochemistry of the phosphate groups in the DNA biomolecule. Note two possible configurations (*pro-R* and *pro-S*) in the P=O bound and the negatively charged oxygen atoms. DNA is a negatively charged biomolecule.

Since Watson and Crick’s three-dimensional model of DNA [24] and related studies [25,26,27,28,29,30], many efforts and progress were made to provide a deeper understanding on its 3D arrangement and conformation [31,32,33,34]. A double helix formation is quite common in DNA structures. Nevertheless, RNA also displays double helix configuration (in its secondary structure) in some circumstances, such as in gene silencing. DNA does not exist as a single three-dimensional structure, but rather can adopt different conformations which are defined both locally and macroscopically by different structural parameters. Basically, DNA is found in three different forms: B-DNA (most common and right-handed orientation), A-DNA (right-handed orientation) and Z-DNA (rare and left-handed orientation) [35,36,37]. It is worth noting that chirality is intrinsically present in the DNA structure both at the molecular and at the supramolecular level. Stereogenic centers can be found in both ribose (RNA) and 2-deoxyribose (DNA) sugar moieties, whose configuration is important in the overall RNA or DNA structure. The chirality concerning DNA has been recently reviewed. [38] Phosphate backbone also has an important role in DNA structure. Phosphate groups are negatively charged and two diastereotopic oxygen atoms of each phosphate group have different chemical and spectroscopic properties (Figure 2). Binding of a chiral guest molecule inside the chiral cavity of a specific host can generate enantioselective responses from their fluorophores. [39]

The different properties of the phosphate group depends on its configuration, especially because they display a major role in the interaction of DNA with different species. The P=O double bond display two heterotopic faces (*pro-R* and *pro-S*). For instance, the mechanism of the *Escherichia coli* DNA T:G mismatch endonuclease (*Vsr*) has been shown to interact with DNA through a specific configuration (*pro-R* face) at its hydrolytic site 40]. In the study it was demonstrated that *Vsr* carries out a hydrolytic reaction with inversion of the configuration at the prochiral face. 

Another important study was carried out by Chaires *et al.*, who used both daunorubicin enantiomers [41]. The (-)-daunorubicin isomer was a newly synthesized enantiomer of the anticancer drug (+)-daunorubicin. Both isomers were tested with left- and right-handed DNA. (+)-Daunorubicin bound selectively to right-handed DNA, whereas the other enantiomer ligand bound selectively to left-handed DNA. Further, binding of the enantiomeric pair to DNA was clearly chirally selective. Moreover, the authors found that each of the enantiomers acts as an allosteric effector of DNA conformation. 

A remarkable feature of the DNA biomacromolecules is that there are several reactive sites uniquely displayed on the surface of the double-helix, depending on the sequence. For instance, in the minor groove of DNA, the N2 amino group of guanine base is particularly susceptible to drug action. The binding specificity of many drugs to DNA often involves the recognition of guanine base in the minor groove through the hydrogen bonding interactions of the exocyclic N2 amino group. In fact, as it has been reviewed, many drugs alkylate to this site [42]. However, the above mentioned amino group is often a steric hinderance that decreases the affinity of groove binders to GC-rich grooves. The N3 atom of both guanine and adenine in the minor groove is also a favorable target for drug action. Finally, the N7 atom of guanine in the major groove is the most reactive site in DNA, onto where many metal ions and alkylating agents attack [42]. 

## 3. Principles of intercalation

**Figure 3:**
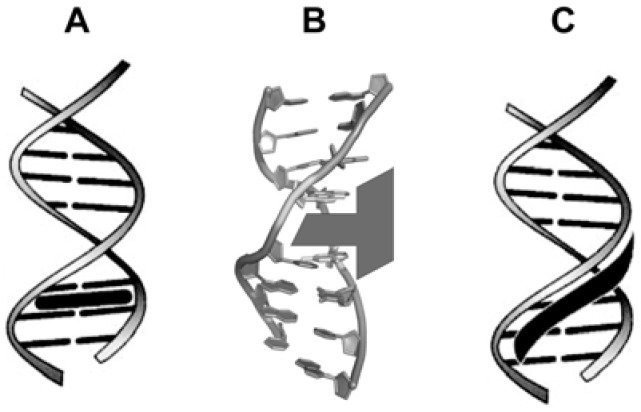
(a) Generic intercalation representation (figure based on a previous report [48]); (b) dsDNA and a schematic representation of a general intercalating agent (figure based on a previous report [49]). Note the intercalation in the major groove of the dsDNA structure; (c) Generic groove binding representation (based on a previous report [48]).

Intercalation into DNA (insertion between a pair of base pairs) is a very important process, especially with regards to the function of many anticancer drugs. In a very important recent article on the subject, Mukherjee *et al.* have pointed out that: “*Despite its importance, a detailed mechanistic understanding of this process at the molecular level is lacking*” [43]. At this point it is important to point out that many chemical species can bind covalently and non-covalently to DNA. Herein, we rather focus in non-covalent species. These specific host-guest interactions may have some consequences as a result of DNA intercalation by exogeneous molecules, such as a significant modification of the DNA structure [44] that may result in a hindered or suppressed function of the nucleic acid in physiological processes [45,46,47]. Furthermore, two common binding modes are observed for these small molecules: these are intercalation (Figure 3a, Figure 3b) or groove-binding (Figure 3c). Intercalation results from insertion of a planar aromatic substituent between DNA base pairs, with concomitant unwinding and lengthening of the DNA helix (this will be discussed later). Groove binding, in contrast, does not perturb the duplex structure to any great extent. Groove-binders (not covered in the current manuscript) are typically crescent-shaped, and fit into the minor groove with little distortion of the DNA structure. 

Intercalators have been properly defined elsewhere. Barton *et al.* define intercalators as: “*…small organic molecules or metal complexes that unwind DNA in order to π-stack between two base pairs*” [49]. The intercalators are oriented parallel to the base pairs, commonly π-stacking in the major groove, although some bindings seem to occur preferentially in the minor groove of DNA [50]. In a dsDNA helix, the nucleic bases are located in an almost coplanar arrangement, which allows planar aromatic molecules to intercalate between two base pairs [51]. When intercalated, it is possible to note π-stack interactions (intercalated moiety), hydrogen-bonding, van der Waals interactions, hydrophobic interactions and steric hindrance effects. In a succinct form, a combination of coulombic, hydrophobic, steric forces and DNA sequence influence the mode of binding which depends on the structure of the agent used [52]. It is important to highlight that upon intercalation, the intercalator causes a distortion on DNA structure. In general, the angle of the phosphate groups change (opening) allowing for the intercalation. The unwinding of the double strand leads to a lengthening of the helix by approximately 3.4 Å, which causes a conformational change of some sugar moieties involved [48,53]. As a consequence of the intercalation, the so-called “neighbor exclusion principle” takes place. This principle determines that after intercalation of a structure, the access of another intercalator to the binding site next to the neighboring intercalation pocket is now hindered, and it does not occur [54]. This fact is relatively obvious since an intercalation results in significant local DNA structural changes [55], which means that deep alterations in the nucleotide secondary structure occur [56,57]. 

If the probe is a cationic organic dye, normally the propensity of this small (organic or organometallic) molecule to bind to DNA is enhance, mostly via interaction of the positive charge with the phosphate backbone in the double-strand DNA macromolecules [44]. Actually, cationic species are the most used as fluorescent intercalators, despite the fact that some neutral intercalators are also used for many different purposes. When using cationic intercalators, one observes a significant electrostatic contribution to the binding energy for molecules with a predominantly positive electrostatic potential (charged or, in some cases, highly polar intercalators), but this varies significantly with sequence, and somewhat with the twist angle, despite the fact that electrostatic binding energy is also unlikely to be a major determinant of the twist angle, as its variation with angle is modest for most intercalation phenomena [58]. Extensive theoretical studies have indicated that, in fact, the dispersion energy contributes mostly to the overall energy of the intercalation complex [59]. 

A complete characterization of DNA binding agents requires that their mode of binding to DNA be established. Actually, it may be a hard task to be performed [60]. Experiments such as spectrophotometric and/or spectrofluorimetric titrations or fluorescence polarization measurements are very useful in order to help the scientists to elucidate the general binding interaction between the guest molecule and DNA [61]. However, it is important to understand that these techniques can not be used for the unambiguous determination of the guest binding mode. Moreover, it was demonstrated that a combination of those experiments associated with viscosimetric titrations and the determination of a fluorescence resonance energy transfer (FRET) may serve as a reliable tool to determine the binding mode of the guest molecule [61]. NMR experiments (^1^H-, ^13^C- and ^31^P-) [62,63,64,65,66,67], theoretical calculations [68,69,70], calorimetric methods [71,72,73], circular and linear dichroism [74,75,76,77,78], X-ray diffraction [79,80,81], and other methods [82] are also extremely useful to determine the binding mode between the guest molecule and dsDNA and some thermodynamic parameters. Additionally, in order to approach a detailed understanding of the molecular forces that drive these interactions, the importance of obtainig thermodynamic information was described [83,84]. 

## 4. Synthesis, intercalation of small fluorescent molecules and possible applications

Efficient synthetic methodologies and the understanding on how fluorescent molecules can be intercalated are major drawbacks to be overcome in the development of nucleic acid technology [85]. As a matter of fact, the need for a deeper understanding to tune some photophysical properties of small fluorescent molecules is a major concern to keep developing light technology [86]. Rational design and the synthesis itself may become the worst problems during the research. Many C-C, C-heteroatom cross-coupling reactions protocols are available nowadays [87,88,89,90,91,92,93,94,95]. Nevertheless, sometimes the obtained yields are extremely low. This class of reactions constitute a direct, elegant, fundamental and mostly used tool to a straight π-extension of an appropriate intermediate. On the other hand, efficient catalysts are under development by many research groups, mainly to promote cross-coupling reactions faster, cleaner and with higher yields. Nevertheless, sometimes it is necessary to perform syntheses with many steps to achieve the desired intercalator. In this sense, interesting works are described in the literature, and at this point, we intend to overview some selected work. Of course, the subject is far from being fully covered, and many other works on the topic are available in the literature. 

**Scheme 1:**
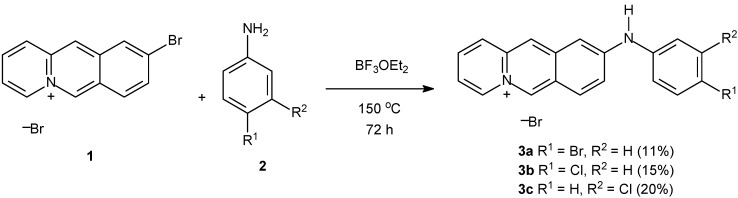
Synthesis of *N*-arylamino-acridizinium derivatives.

One example is a the excellent study of Ihmels *et al.* using *N*-aryl-9-amino-substituted acridizinium derivatives [96] (Scheme 1). These derivatives were directly synthesized upon treating **1** with aniline derivatives (**2**) at 150 °C. Note that, despite the synthesis being direct, yields are not so high as we wished. However, the obtained yields are very good for this specific reaction, which is a hard task to accomplish. 

Their novel fluorescence probes, whose interaction with DNA and proteins could be monitored by absorption and emission spectroscopy, offered promising properties for DNA detection. In a further work [97] the same group used spectrophotometric titrations and circular dichroism to conclude that acridizinium derivatives probes are almost insensitive to the changes in the polarity of the medium, but with a pronounced susceptibility to the rigidity of the environment. In some fluorescent probes tested, they noted intercalation with a coplanar orientation of the chromophore plane relative to the plane of the DNA bases. 

**Scheme 2:**
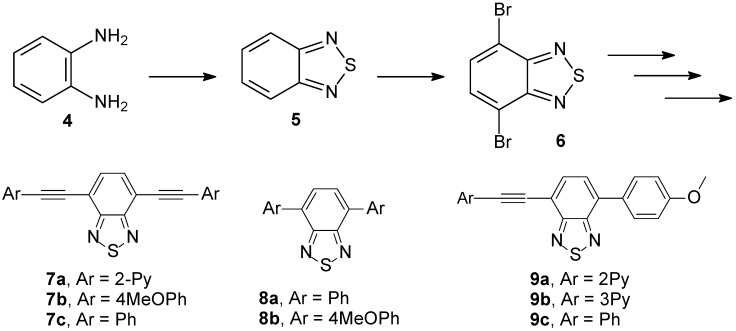
Synthesis of fluorescent BTD derivatives.

Other example is the work reported by our group using neutral and polar 2,1,3-benzothiadiazole-containing chromophores. These fluorescent 2,1,3-benzothiadiazole derivatives proved to be excellent light-up probes for selective dsDNA detection acting as intercalating agents (Scheme 2) [98]. 

**Scheme 3:**
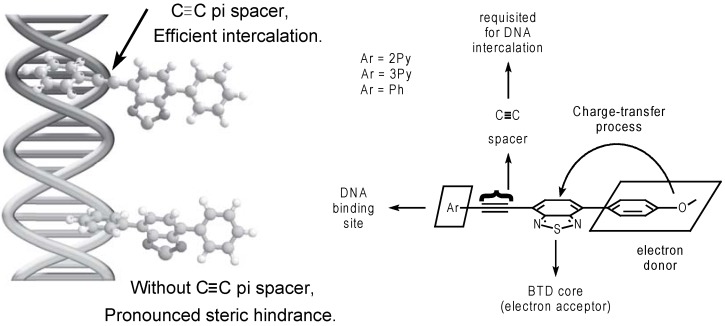
Intercalation model proposed for 2,1,3-BTDs derivatives. The intercalation requires the presence of a C≡C spacer at least on one side of the molecule.

Compounds **7a-c**, **8a,b** and **9a-c** were synthesized with high overall yields using Sonogashira and Suzuki cross-coupling reactions. Suzuki cross-coupling reactions required the use of Dupont’s catalyst [99] in order to achieve higher yields in the reaction [100,101]. The use of unsymmetrical dyes **9a-c **gave better results for the spectroscopic selective detection and quantification of DNA. An intercalating model (Scheme 3), explaining the molecular architecture and the principles of stabilization in the excited state, was proposed. 

**Scheme 4:**
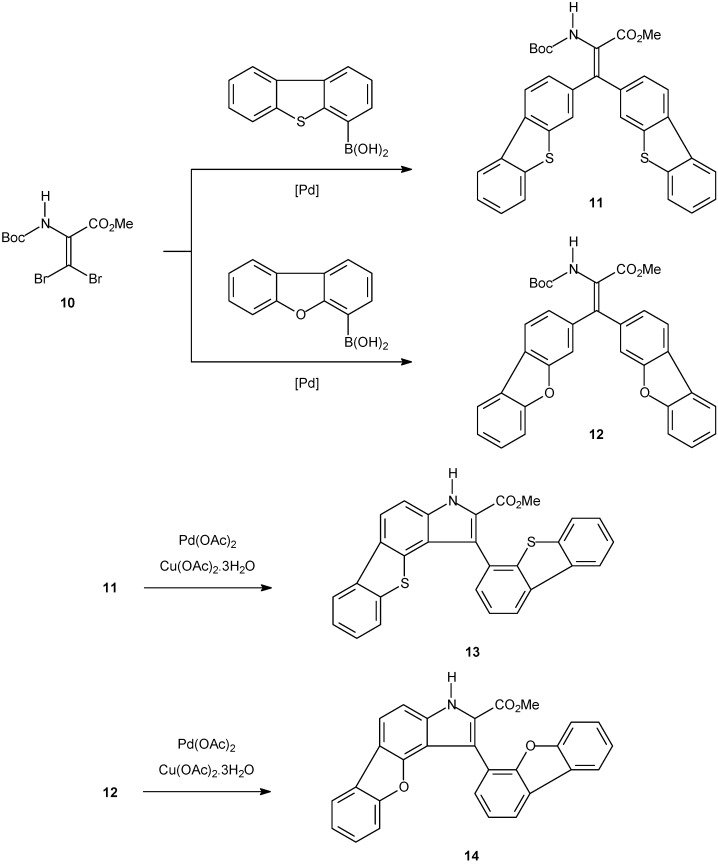
Synthesis of fluorescent benzothienoindole and a benzofuroindole tested as intercalating agents.

A benzothienoindole and a benzofuroindole were synthesized with high yields (Scheme 4) and studied as intercalating agents [102]. The results of spectroscopic and electrochemical studies revealed that benzothienoindole is the more intercalative compound and has higher affinity for DNA. 

Two new tetracyclic neutral and highly polar compounds were synthesized by an intramolecular C-N metal-assisted cyclization. The desired dyes were prepared by a bis-Suzuki coupling of a β,β-dibromodehydroalanine derivative and dibenzothien-4-yl and dibenzofur-4-yl boronic acids. 

The binding constants between salmon sperm dsDNA and both benzothienoindole **13** and benzofuroindole **14** were determined as (3.8 ± 0.3) x 10^5^ and (1.3 ± 0.1) x 10^5^, respectively. The combination of spectroscopic and electrochemical methods was helpful in the understanding of the interaction between dsDNA and the tested dyes. Intercalation was the preferred binding mode. Additionally, the experiments helped to determine the recognition of DNA sites, and also to promote novel rational design of drugs for chemotherapy applications. 

**Scheme 5:**
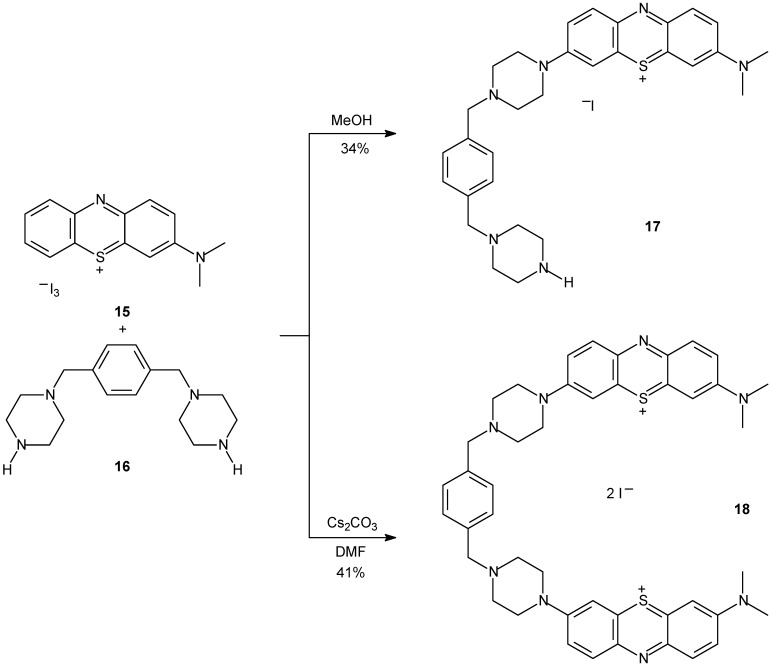
Synthesis of cationic fluorescent derivatives **17** and **18**.

Chromophore systems consisting of one or two phenothiazine rings covalently attached to a bis-piperazinexylene chain were synthesized (Scheme 5) and evaluated as DNA intercalating agents [103]. In the presence of DNA, these compounds were shown to monointercalate in their deaggregated forms and to strongly absorb red light wavelengths (650-700 nm). 

Fluorescent systems were obtained in good overall yields. Interestingly, the cationic compounds **17** and **18** acted as DNA photocleavage agents. When bound to DNA, they generate significant levels of duplex stabilization and exhibit strong absorbance ranging between 600 and 800 nm, the therapeutic window required for photodynamic cancer therapy. Since it was observed that, at micromolar dye concentrations, robust levels of DNA photocleavage are produced under near-physiological conditions of temperature and pH (22 °C and at pH 7.0), the authors suggest that their systems may serve as a good starting point for the development of new phototherapeutic agents. 

**Scheme 6:**
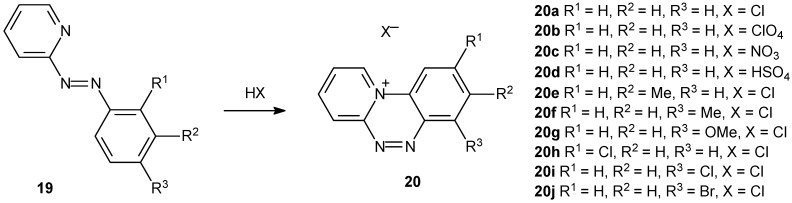
Synthesis of a novel family of planar triazinium fluorescent salts.

A novel family of planar triazinium fluorescent salts were synthesized (Scheme 6) with good yields (60-65%) and tested as DNA photointercalators [104]. The synthesis is concise and the methodology new. The novel compounds exhibited good water solubility. 

The authors concentrated their tests using compound **20a** and showed that the fluorescent intensity decreased steadily nearly by 25% upon DNA addition. The relative binding constant was determined and the value was K = 2.6 x 10^4^ M^-1^). Studies *in vitro* and *ex vivo* have confirmed that compound **20a** binds to DNA strongly even in the nanogram range. The authors suggested that their compounds might be relevant to the biomimetric approach of *in vitro* DNA damaging using chemical nucleases, which is a topic of interest for elucidating the genetic mechanisms of the natural enzymes involved in DNA scission, repair and signal transduction. 

## 5. Clinical applications

Yang and Wang precisely wrote that: “*it is worth mentioning that while DNA is considered the ultimate cellular target of many anticancer drugs, other cellular targets are also possible*” [42]. This means that DNA is a very promising target, but not the only one. The discovery and development of novel therapeutic intercalators agents for the treatment of malignancy are some of the most important goals in modern medicinal chemistry. A very interesting group in cancer therapy comprises molecules that target directly dsDNA. A very good (mini-)review on DNA intercalators in cancer therapy has been recently published [21]. However, the topic is far from being exhausted. Clinical applications are a topic of high importance for many different purposes, specially in life sciences. Based on some recently published results, new drugs can be developed, new therapies applied, new process discovered. For all these reasons, the understanding on how different intercalators are interacting with dsDNA is a challenging task of paramount importance. Once more, the drawback of an appropriate synthetic methodology to achieve the molecular target is a problem that we still need to deal with. However, many groups have made much progress in the synthesis and application of some intercalators. It is also important to point out that many intercalators do not display therapeutic properties, but may cause some damages to DNA and/or to the organism. These structural modifications upon binding may lead to the retardation or inhibition of transcription and replication, and DNA intercalators may be mutagenic. The genotoxicity of non-covalent interactions have been already reviewed [105,106]. In spite of this, many researches are promising. In addition, controlled mutation may be desirable. At this point, we disclose some selected works to highlight the challenges and progress already made. 

**Scheme 7:**
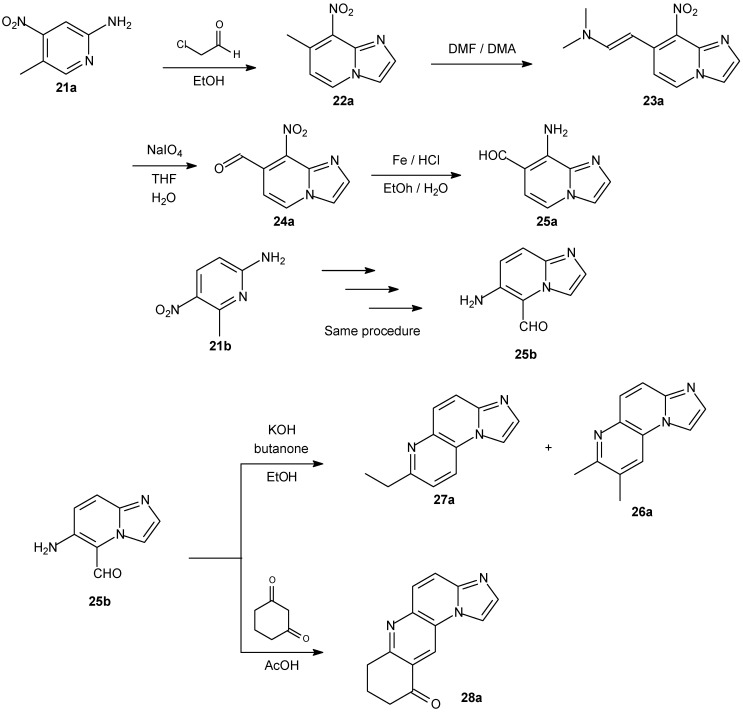
Synthesis of a novel family of planar triazinium fluorescent derivatives.

Some imidazopyridine derivatives were synthesized (Scheme 7) and evaluated for their antitumor activity in the NCIs *in vitro* human tumor cell line screening panel [107]. It should be noted that, although the synthesis was not direct, the authors achieved the desired compounds with reasonable yields. Actually the methodology allowed them to obtain 20 different angular imidazonaphthyridinic derivatives.

They accessed the antiproliferative activities on four different cell lines along with their DNA-intercalating properties and their topoisomerase inhibition power. Interestingly, they highlighted the direct intercalation of the drugs into DNA strands by electrophoresis on agarose gel. Their compounds are a new class of DNA-recognizing derivatives.

**Scheme 8:**
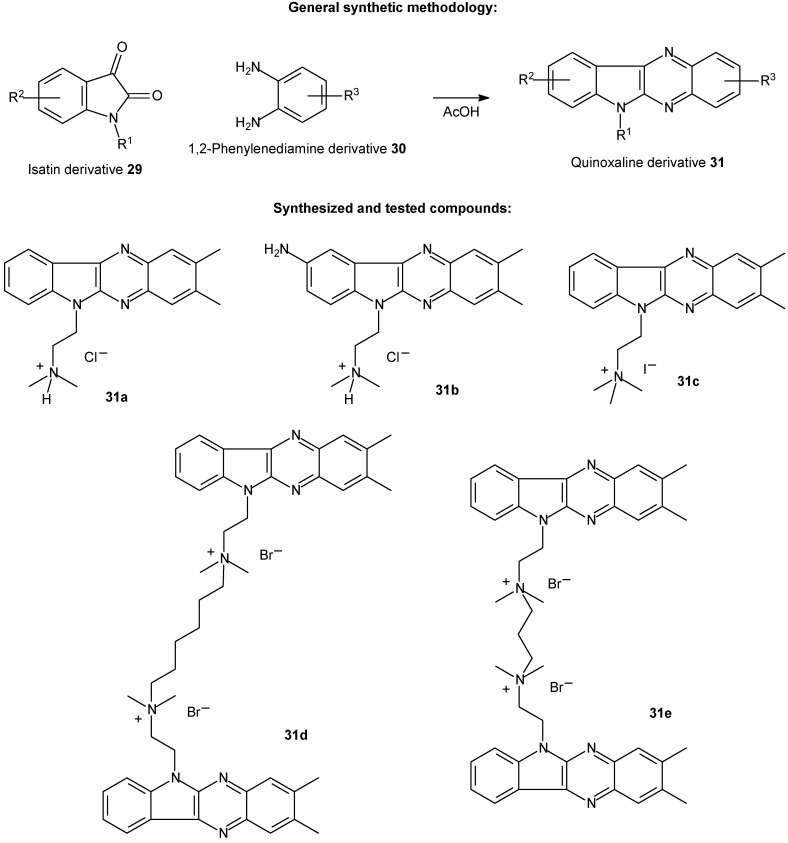
Synthesis of a novel family of quinoxalines derivatives.

Other recently published and important work was conducted by J. Bergman and co-workers [108]. The authors performed the synthesis of quinoxalines derivatives (Scheme 8) and tested their antiviral activity. 

The new systems were easily prepared by condensation of the isatin derivative **29** and the appropriate 1,2-phenylenediamine **30** in glacial acetic acid. Isatin derivatives can be directly prepared, as discussed in previous reports [109,110]. It is worth noting that the synthetic methodology used by the authors was very efficient and straightforward with very good yields. 

Compound **31b** was virtually nonfluorescent, both in the presence and absence of DNA. However, in the presence of dsDNA, the light-up effect was pronounced for all other compounds (**31a** and **31c-e**). All λ_max_ (emission wavelengths) were above 450 nm. Using fluorescence and circular dichroism, it was possible to conclude that the new compounds bind strongly but noncovalently to DNA in an intercalative mode. Furthermore, they are found to have equally high binding constants as already established DNA drugs and dyes. It is interesting to highlight that the molecular guests also displayed AT-specificity, which is a property shared with some of the DNA drugs and dyes. This fact is potentially useful for targeting viral genomes that are especially AT-rich. 

**Scheme 9:**
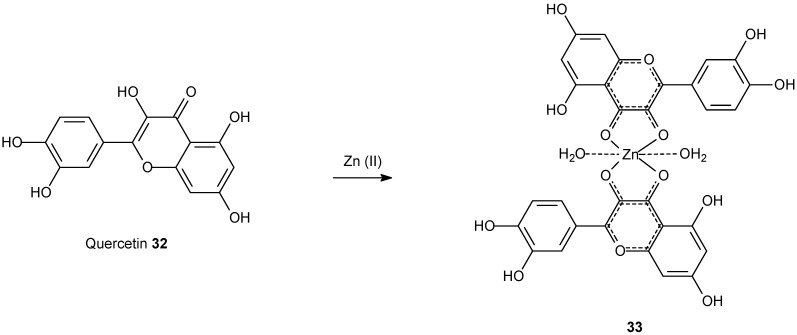
Quercetin zinc(II) complex derivative.

A quercetin zinc(II) complex derivative (Scheme 9) has been tested *in vitro* using three tumor cell lines (HepG2, SMMC7721 and A549), and showed significant cytotoxicity against three tumor cell lines. [111] Moreover, Hoechst33258 staining showed that HepG2 cells underwent the typical morphologic changes of apoptosis after exposure to the complex. 

Quercetin is a bioflavonoid widely found in fruits and vegetables and has been reported to exert multiple biological effects as antioxidant and antitumor agent [112]. The synthesis of the zinc complex is direct and easy to be performed. 

**Scheme 10:**
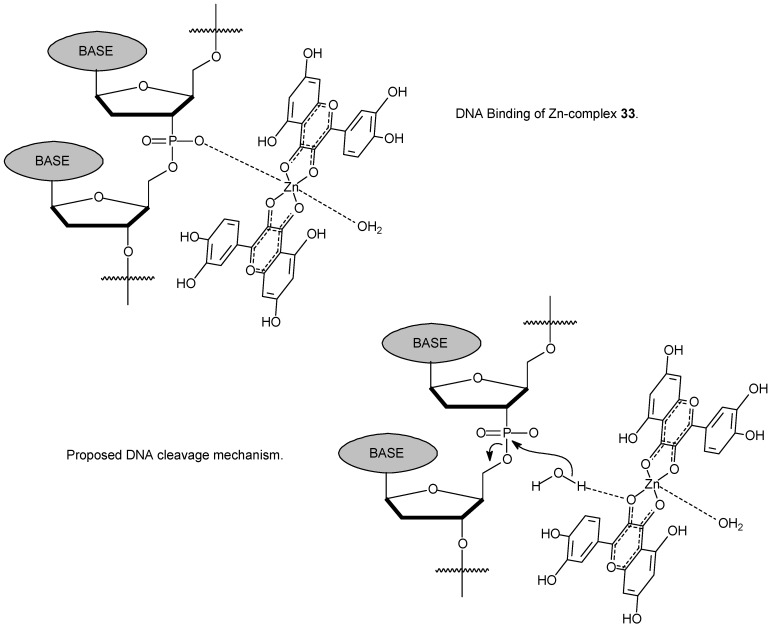
Proposed intermediate of DNA binding with quercetin zinc(II) complex **33 **and proposed DNA cleavage mechanism. The fluorescent complex helps in the hydrolysis process on the phosphate group.

In the study it was concluded that complex **33** could interact with DNA via intercalation mode. Equally, compound **33 **displayed a significant inhibition to growth and proliferation of tumor cells (HepG2, SMMC7721 and A549) in a dose- and time-dependent manner. Also, IC_50_ values provided by the complex are much lower than that of quercetin **32** alone. The complex is probably inducing apoptosis of tumor cells. Interestingly, molecular modeling was performed revealing that the system **33** probably binds preferentially in a GC region. On the basis of those results, a model of DNA cleavage induced by complex **33** was proposed, as shown in Scheme 10. The mechanism of action of the zinc complex during the cleavage process can be clearly depicted from the scheme. 

## 6. Conclusions and Trends

There are still many drawbacks to be overcome and progress to be made in the chemistry of DNA photointercalators. Considering the increasing contribution of cancer to the overall mortality rate, a more rational design and application of novel DNA intercalators, with both higher efficiency and selectivity, constitutes an urgent task in medicinal chemistry. It is more than likely that DNA-binding properties of an intercalator new drug may play a key role in different chemotherapies. As a natural consequence, the understanding of the association of a photointercalator and dsDNA is among the most significant contributions to the overall struggle against many diseases. The spectroscopic techniques cited in this review may provide all data necessary to a better comprehension of the intercalation mode of small guest molecules and dsDNA. 

In a general way, chemical modifications are made to the core structure of traditional intercalators, but a more rational design is still challenging many research groups. This is mainly due to their vast structural diversity and the problems associated with the complete characterization of the binding mode of new structures. In the case of small molecular fluorophores, this association may not be very clear, mainly because of the great diversity of the possible resulting structures. Higher selectivity, sensitivity, shorter assay times and greater simplicity in performing the assay are trends that must be taken into account in the design of new photointercalators that may be commercially viable. Perhaps, appropriate synthetic methodologies and good overall yields are some of the major problems to be solved. A case where the synthesis is performed in multi-step reactions and the yields are not so high is not rare. 

For clinical proposes, before interacting with DNA, the intercalating agents must overcome many barriers, including metabolic pathways and the cytoplasmic and nuclear membranes. As such, clinical failure of most intercalator drugs can be attributed more to pharmacokinetics than to pharmacodynamics [113]. However, despite many possible problems such as toxicity, nonselectivity, and costs, at the present time, DNA intercalators are among the most important and promising therapeutic agents to treat many diseases such as cancer. 
